# The Use of Information and Communication Technology–Based Self-management System DialBeticsLite in Treating Abdominal Obesity in Japanese Office Workers: Prospective Single-Arm Pilot Intervention Study

**DOI:** 10.2196/40366

**Published:** 2022-11-28

**Authors:** Yuki Kawai, Kayo Waki, Satoko Yamaguchi, Tomomi Shibuta, Kana Miyake, Shigeko Kimura, Tsuguyoshi Toyooka, Ryo Nakajima, Kazushi Uneda, Hiromichi Wakui, Kouichi Tamura, Masaomi Nangaku, Kazuhiko Ohe

**Affiliations:** 1 Department of Biomedical Informatics Graduate School of Medicine The University of Tokyo Bunkyo-ku, Tokyo Japan; 2 Department of Medical Science and Cardiorenal Medicine Yokohama City University Graduate School of Medicine Yokohama Japan; 3 Department of Diabetes and Metabolic Diseases Graduate School of Medicine The University of Tokyo Bunkyo-ku, Tokyo Japan; 4 Department of Prevention of Diabetes and Lifestyle-Related Diseases Graduate School of Medicine The University of Tokyo Bunkyo-ku, Tokyo Japan; 5 Department of Healthcare Information Management The University of Tokyo Hospital Bunkyo-ku, Tokyo Japan; 6 Healthcare and Medical Business Smart-Life Solutions Department NTT DOCOMO, Inc Chiyoda City Japan; 7 Division of Nephrology and Endocrinology Graduate School of Medicine The University of Tokyo Bunkyo-ku, Tokyo Japan

**Keywords:** abdominal obesity, self-management, telemedicine, mobile phone

## Abstract

**Background:**

Making lifestyle changes is an essential element of abdominal obesity (AO) reduction. To support lifestyle modification and self-management, we developed an information and communication technology–based self-management system—DialBeticsLite—with a fully automated dietary evaluation function for the treatment of AO.

**Objective:**

The objective of this study was to evaluate the preliminary efficacy and feasibility of DialBeticsLite among Japanese office workers with AO.

**Methods:**

A 2- to 3-month prospective single-arm pilot intervention study was designed to assess the effects of the intervention using DialBeticsLite. The information and communication technology system was composed of 4 modules: data transmission (body weight, blood pressure, blood glucose, and pedometer count); data evaluation; exercise input; and food recording and dietary evaluation. Eligible participants were workers who were aged ≥20 years and with AO (waist circumference ≥85 cm for men and ≥90 cm for women). Physical parameters, blood tests, nutritional intake, and self-care behavior were compared at baseline and after the intervention.

**Results:**

A total of 48 participants provided completed data for analysis, which yielded a study retention rate of 100%. The average age was 46.8 (SD 6.8) years, and 92% (44/48) of participants were male. The overall average measurement rate of DialBeticsLite, calculated by dividing the number of days with at least one measurement by the number of days of the intervention, was 98.6% (SD 3.4%). In total, 85% (41/48) of the participants reported that their participation in the study helped them to improve their lifestyle. BMI, waist circumference, and visceral fat area decreased significantly after the intervention (*P*<.001). In addition, the daily calorie intake reduced significantly (*P*=.02). There was a significant improvement in self-care behavior in terms of exercise and diet (*P*=.001).

**Conclusions:**

Using DialBeticsLite was shown to be a feasible and potentially effective method for reducing AO by providing users with a motivational framework to evaluate their lifestyle behaviors.

## Introduction

### Background

Obesity is a global public health problem because of its high prevalence and associated morbidity and mortality [1]. In particular, abdominal obesity (AO), which is defined by intra-abdominal fat deposition, is associated with an increased risk of multiple chronic diseases such as cardiovascular disease, atherosclerosis, and type 2 diabetes.

Evidence from preceding studies strongly supports the benefits of lifestyle changes—diet and exercise—as effective means to reduce AO [2-4]. However, behavioral interventions can be difficult to achieve in wide-scale clinical practice because of limited resources and inadequate professional support [5,6]. In addition, it is often challenging to maintain obesity reduction with behavioral lifestyle changes over a long period [7,8].

As such, interventions that use information and communication technology (ICT) show great promise in terms of effectiveness and scalability. Given that weight loss has been shown to improve through the use of ICT interventions, the authors of this study sought to investigate the utility of ICT tools as a means of providing behavioral intervention to those with AO [9,10]. We previously reported that the self-management ICT system “DialBetics” improved glycemic control in patients with type 2 diabetes [11]. The system was shown to be a feasible and an effective tool for improving hemoglobin A_1c_ (HbA_1c_) by providing patients with real-time support based on their measurements. Furthermore, we have developed the self-management ICT system “DialBeticsLite” to provide a similar approach to addressing AO. The upgrade from DialBetics to DialBeticsLite includes fully automated instant calculation and evaluative feedback on nutrient intake, which replaced the manual calculation of nutritional values by dieticians, which took 1 to 2 days. This intervention assists patients to practice lifestyle self-management through daily self-monitoring of blood pressure (BP), blood glucose, pedometer count, body weight (BW), exercise, and diet.

Systematic reviews reported that the use of mobile health (mHealth) showed a modest short-term effect on BW and BMI in adults with obesity [12], and internet-based interventions have a significant and promising effect on waist circumference (WC) change [13]. Nevertheless, these studies did not further investigate the effect of mHealth lifestyle interventions on visceral fat area among the population with AO. Excess visceral fat area is a well-known risk factor for the development of diabetes mellitus and onset of cardiovascular disorders [14].

### Goal of This Study

In this study, we performed a pilot study to test DialBeticsLite in a population with AO. This study aimed to evaluate the effects of a lifestyle intervention using ICT-based self-management systems on physical and metabolic parameters, including visceral fat area, as well as self-care behavioral parameters in participants with AO.

## Methods

### Design

A prospective single-arm pilot study using the intervention DialBeticsLite was conducted.

### Ethics Approval

The study was approved by the institutional review board of the Graduate School of Medicine, the University of Tokyo (approval numbers: 3283-(10) and 11475) and was carried out in accordance with the Declaration of Helsinki.

### Participants

Japanese office workers who were aged ≥20 years; with AO (WC ≥85 cm for men and ≥90 cm for women), which was determined at routine health checkups held at work sites in the year before this study; and willing to use the ICT system were eligible for the study. The WC cutoff points were chosen based on the recommendation of the Japanese Committee of the Criteria for Metabolic Syndrome, which adopts cutoffs of WC ≥85 cm for men and ≥90 cm for women [15]. Those who received any medications for the treatment of hypertension, dyslipidemia, or diabetes were excluded. The applicants provided written informed consent before study participation. As literature that focuses on mHealth interventions for visceral fat area reduction is scarce, we have no information on the effect size for sample size calculation. Therefore, we recruited at least 55 patients as a suitable sample size for a pilot feasibility study based on recommendations in the literature [16].

### Interventions

#### Educational Group Session

Before using DialBeticsLite, the participants attended an educational group session at the University of Tokyo Hospital. The research team, consisting of nurses, diabetologists, and dietitians, gave a 40-minute lecture about diet and exercise therapy. The research team trained all the 48 participants in the use of DialBeticsLite and ensured that they could use the app and devices competently. The participants were college educated and used cell phones for calls and SMS text message exchanges daily; none of them had difficulties in using DialBeticsLite and measurement devices. They were allowed to contact the research team if they encountered any issues during the use of the app and devices.

#### Use of DialBeticsLite

**Figure 1:**
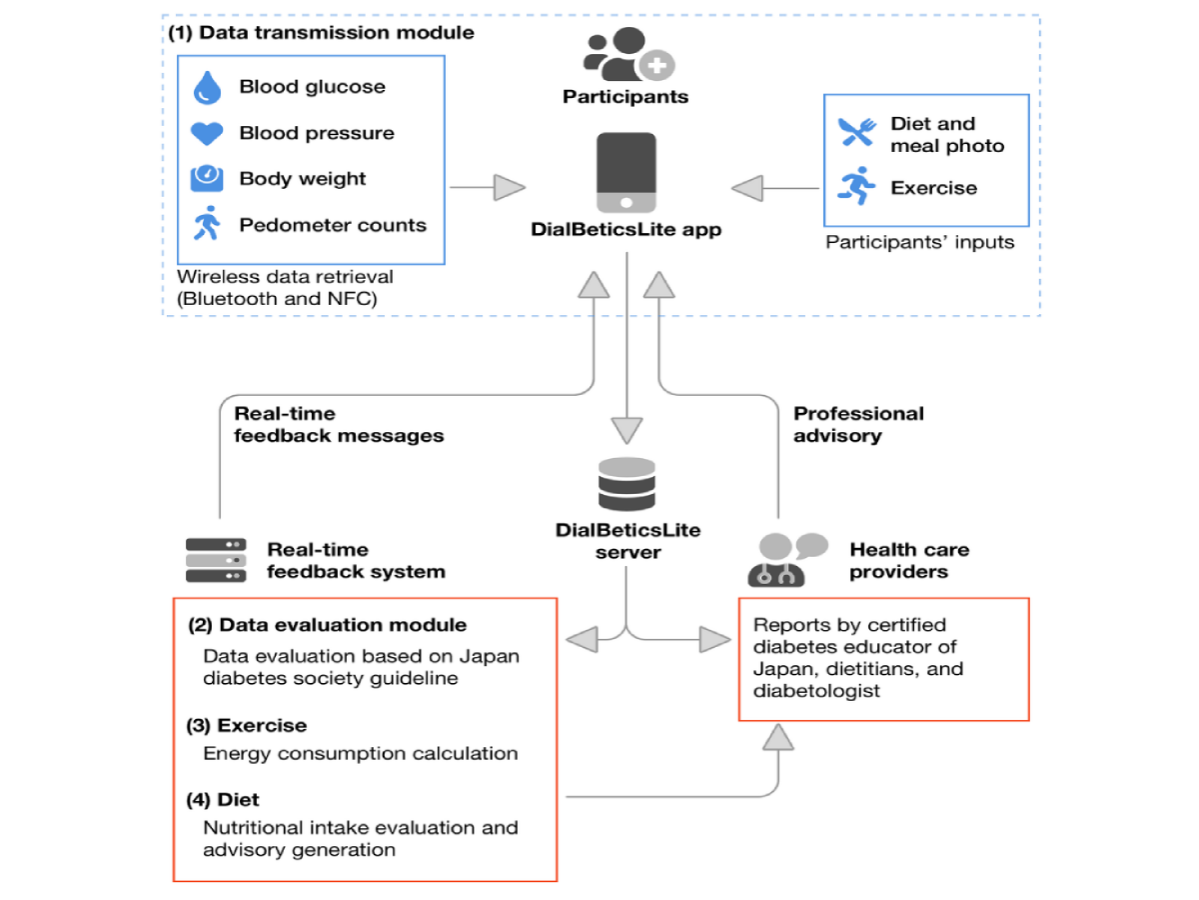
Data transmission module, data evaluation module, exercise, and nutritional evaluation function of DialBeticsLite. NFC: near field communication.

The participants used DialBeticsLite for 2 or 3 months (61 days, March 1 to April 30, 2017, for the participants from company X and 93 days, May 22 to August 22, 2017, for those from company Y). Both companies had different time availabilities for study participation owing to unavoidable job circumstances. The DialBeticsLite system is outlined in Figure 1.

At home, the participants measured their blood glucose, BP, and BW upon waking in the morning and blood glucose and BP at bedtime. They wore a pedometer and measured their number of daily steps and the calories consumed during the activity. Blood glucose was measured by the participants who were willing to do so. The participants transferred measured data from each device to the smartphone by near field communication or Bluetooth; all data were sent to the server directly following measurement, excluding pedometer counts, which were sent at least once a day at bedtime.

The data were automatically evaluated according to the Japan Diabetes Society guideline’s target values [17]: optimally, glucose level below 110 mg/dL before breakfast and below 140 mg/dL at bedtime and BP below 125/75 mm Hg. The number of daily steps was evaluated according to the Japanese official physical activity guidelines for health promotion, with a target of ≥8000 steps per day [18]. DialBeticsLite then determined whether each reading satisfied the guidelines [17,18] and immediately sent those results to each participant’s smartphone.

Dietary information (food names and quantity of each meal with photos) and exercise information (type of exercise and its duration) not counted by a pedometer were inputted by the participants and sent to the server; specific advice on diet modification was sent back to each participant immediately after the input. The participants were provided with a set of devices for the study: a smartphone (Galaxy Note3 SC-01F [Samsung]), a Bluetooth-enabled BP monitor (HEM-7271T [Omron]) and scale (HBF-255T [Omron]), a near field communication–enabled glucometer (MS-FR201B [Terumo]), and a pedometer (MT-KT02DZ [Terumo]).

The system triggered alerts if a participant recorded no data for more than 3 days; the alerts could be checked on the administrator screen. The nurse emailed (after 1 week of inactivity) or called (after 2 weeks of inactivity), encouraging the participants to restart measurements. If a participant recorded no data (measured data, food, or exercise) for 3 weeks, we designated the participant as a dropout.

#### Report From Health Care Providers

The participants received a specific feedback report from the research team at the end of the study. The nurses and dietitians of the study team drafted the *Advice From Healthcare Providers* and physicians of the team reviewed and approved them before distributing them to the participants. The report was based on measured data, and recorded data were provided, including measurement rates, target achievement rates, averages of measured values, lifestyle evaluations, and suggestions for further improvement (Multimedia Appendix 1).

### Data Collection and Outcomes Measure

#### Total Measurement Rate, Food Recording Rate, and Exercise Input Rate

We assessed the total measurement rate, food recording rate, and exercise input rate of the DialBeticsLite users in this study. The total measurement rate, measurement rate for each parameter (blood glucose, BP, BW, and pedometer counts), exercise input rate, and food recording rate were each calculated. The total measurement rate was calculated by dividing the number of days with at least one measurement by the number of days of the intervention (ie, 61 or 93 days). Similarly, the measurement rate for each parameter was calculated by dividing the number of days on which that parameter was measured by the total number of intervention days.

#### Changes in Physical and Metabolic Parameters

All participants underwent physical measurements and blood tests at the time of the educational group session, which immediately preceded the start of the study and happened after 2 or 3 months of the use of DialBeticsLite, by a trained nurse or clinical laboratory technician, with the exception of BP. The BP of the patients were measured at home at baseline and after the intervention. The participants’ height, BW, BP, WC, and visceral fat area were measured. Visceral fat area was measured by differentiating visceral fat and abdominal subcutaneous fat using dual bioelectrical impedance analysis, which measures the bioelectrical impedance of the entire abdomen and its surface with a dual current path [19] (DUALSCAN, HDS-2000 [Fukuda Colin]). It has been reported that the estimation of visceral fat area by dual bioelectrical impedance is significantly correlated with that by the gold standard method measured by x-ray computed tomography [20]. We calculated BMI from height and BW. The blood test results included the levels of fasting plasma glucose, HbA_1c_, total cholesterol, triglyceride, and high-density lipoprotein cholesterol.

#### Changes in Behavioral Parameters

To assess the changes in nutritional intake, we asked the participants to fill in a paper-based 7-day meal record at the beginning of the study (within 2 weeks before the use of DialBeticsLite) and the end of the study (within 1 month after the use of the system). The participants recorded the ingredients and their portion size for the food and drinks they consumed for breakfast, lunch, dinner, and in between meal–snacks on a record sheet. The researchers briefed the participants on the correct filling of the dietary record before the study. Dietitians calculated the nutritional intake (total energy [kcal], carbohydrate [g], protein [g], lipid [g], dietary fiber [g], and salt [g]) from the record.

The Japanese-Translated Summary of Diabetes Self-Care Activities (J-SDSCA) measure was included in the questionnaires at baseline and after the intervention to evaluate changes in self-care behaviors [21]. We used only 2 subscales of the J-SDSCA, namely diet and exercise. The J-SDSCA was developed to assess self-care activities for patients with diabetes, but because there is no standardized questionnaire to evaluate self-care activities for people with AO, we used the J-SDSCA for this study.

We compared the mean number of daily steps between the first 7 days and last 7 days of the DialBeticsLite use period. When the participants recorded the number of steps ≥2 times on the same day, the last recorded data were defined as the total number of steps for that day.

#### Usability of DialBeticsLite

We conducted a usability survey for DialBeticsLite after the intervention using the same questionnaire as that in the previous DialBetics study [11]. After the intervention period, the research team informed the participants to attend a wrap-up session in the hospital and asked them to fill out a usability survey. There were 15 statements in this survey, and each statement had a “yes” or “no” option. The research team, which consisted of 1 endocrinologist, 1 cardiologist, 2 nurses, and 2 dieticians, confirmed the face validity of the survey to investigate the usability of DialBeticsLite.

### Statistical Analysis

Data are presented as mean (SD) for parametric variables or median (IQR) for nonparametric variables. Changes in physical and metabolic parameters, the mean daily intake of energy and each nutrient calculated from the 7-day meal record, and the mean number of daily steps were compared using the paired, 2-tailed *t* test for parametric variables or the Wilcoxon signed-rank test for nonparametric variables. Changes in the J-SDSCA scores were compared using the Wilcoxon signed-rank test. Changes in the level of AO were compared using the McNemar test. *P* values of <.05 were considered statistically significant. Statistical analyses were performed using SPSS for Windows (version 25.0; IBM Corp).

## Results

### Overview

A total of 56 individuals from 2 companies participated in this study. After completion of the study, we found that 8 participants had already received medication for hypertension, dyslipidemia, or diabetes; they were excluded from the analyses because they did not meet our eligibility criteria. Finally, 48 participants (n=18, 38% from company X and n=30, 62% from company Y) were included in the analyses. There were no dropouts.

The average participant age was 46.8 (SD 6.8; range 32-62) years, and 92% (44/48) of participants were male. The median WC was 95.0 (IQR 91.0-102.5) cm in men and 98.0 (IQR 91.5-102.3) cm in women. The median visceral fat area was 112.0 (IQR 90.0-147.0) cm^2^.

### Measurement and Recording Rates

The measurement rates are presented in Table 1. The total measurement rate was 98.6% (SD 3.4%). Of the 48 participants, 32 (67%; n=18, 38% from company X and n=14, 29% from company Y) measured blood glucose; the measurement rates of blood glucose before breakfast and at bedtime were 84.5% (SD 24.4%) and 74.4% (SD 27.1%), respectively. The measurement rates of BP before breakfast and at bedtime were 83.9% (SD 20.7%) and 62.2% (SD 32.1%), respectively. The measurement rates of BW, pedometer count, exercise input, and food input were 90.8% (SD 10.7%), 91.1% (SD 16%), 12.5% (SD 19.2%), and 88.5% (SD 20.1%), respectively (n=48).

**Table 1:** The measurement rate of each feature in DialBeticsLite.

Variables		Measurement rates (%), mean (SD)
The total measurement rate (n=48)		98.6 (3.4)
**Blood glucose (n=32)**		
	Before breakfast	84.5 (24.4)
	At bedtime	74.4 (27.1)
**Blood pressure (n=48)**		
	Before breakfast	83.9 (20.7)
	At bedtime	62.2 (32.1)
Body weight (n=48)		90.8 (10.7)
Pedometer count (n=48)		91.1 (16.0)
Exercise input (n=48)		12.5 (19.2)
Dietary record input (n=48)		88.5 (20.1)

### Changes in Physical and Metabolic Parameters

Changes in the physical parameters between baseline and after using DialBeticsLite are presented in Table 2. BW and median BMI significantly decreased from 82.6 (SD 13.1) to 79.0 (SD 12.9) kg (*P*<.001) and 27.0 (IQR 25.5-31.1) to 26.3 (IQR 24.1-29.4) kg/m^2^ (*P*<.001), respectively. WC and visceral fat area also significantly decreased from 95.0 (IQR 91.0-102.5) to 91.7 (IQR 87.0-97.8) cm (*P*<.001) and from 112.0 (IQR 90.0-147.0) to 92.0 (IQR 68.3-113.8) cm^2^ (*P*<.001), respectively. The prevalence of AO was significantly reduced by 13% after using DialBeticsLite (before, n=48 vs after, n=42; *P*=.03).

After using DialBeticsLite, the participants exhibited significant reductions in systolic and diastolic BP, from 131.8 (SD 14.7) to 126.6 (SD 14.3) mm Hg (*P*=.02) and from 88.1 (SD 11.5) to 84.7 (SD 10.2) mm Hg (*P*=.02), respectively. Fasting blood glucose levels and HbA_1c_ significantly declined from 90.0 (IQR 85.0-95.0) to 87.0 (IQR 80.3-93.5) mg/dL (*P*=.01) and from 5.6% (IQR 5.3%-5.8%) to 5.4% (IQR 5.2%-5.6%; *P*<.001), respectively. Total cholesterol lowered from 215.5 (IQR 188.0-234.3) to 194.5 (IQR 175.5-218.0) mg/dL (*P*<.001).

**Table 2:** Changes in physical parameters, blood pressure (BP), glucose, and lipid metabolism before and after the DialBeticsLite intervention.

Variables		Baseline measurements (n=48)	Measurements after the intervention (n=48)	*P* values
**Physical parameters**				
	Body weight (kg), mean (SD)	82.6 (13.1)	79.0 (12.9)	<.001^a^
	BMI (kg/m^2^), median (IQR)	27.0 (25.5-31.1)	26.3 (24.1-29.4)	<.001^b^
	Waist circumference (cm), median (IQR)	95.0 (91.0-102.5)	91.7 (87.0-97.8)	<.001^b^
	Visceral fat area (cm^2^), median (IQR)	112.0 (90.0-147.0)	92.0 (68.3-113.8)	<.001^b^
**Home BP (n=43)**				
	Systolic BP (mm Hg), mean (SD)	131.8 (14.7)	126.6 (14.3)	.02^a^
	Diastolic BP (mm Hg), mean (SD)	88.1 (11.5)	84.7 (10.2)	.02^a^
**Glucose and lipid metabolism**				
	Fasting plasma glucose (mg/dL), median (IQR)	90.0 (85.0-95.0)	87.0 (80.3-93.5)	.01^b^
	HbA_1c_^c^ (%), median (IQR)	5.6 (5.3-5.8)	5.4 (5.2-5.6)	<.001^b^
	Total cholesterol (mg/dL), median (IQR)	215.5 (188.0-234.3)	194.5 (175.5-218.0)	<.001^b^
	HDL^d^ cholesterol (mg/dL), mean (SD)	50.0 (11.3)	48.9 (12.3)	.23^a^
	Triglyceride (mg/dL), median (IQR)	120.5 (78.3-215.3)	110.5 (68.3-165.0)	.06^b^

^a^Paired, 2-tailed *t* test for parametric variables.

^b^Wilcoxon signed-rank test for nonparametric variables.

^c^HbA_1c_: hemoglobin A_1c_.

^d^HDL: high-density lipoprotein.

### Changes in Behavioral Parameters Between Baseline Measurement and Postintervention Measurement

Changes in the nutritional intake based on the 7-day food records are presented in Table 3. The daily energy intake significantly declined from a median of 1953 (IQR 1790-2395) to 1877 (IQR 1690-2153) kcal (*P*=.02). The daily intake of protein and carbohydrates significantly decreased from 71.4 (IQR 63.5-86.4) to 66.4 (IQR 57.2-79.6) g (*P*=.002) and from 227.7 (IQR 198.4-277.6) to 216.3 (IQR 181.6-250.5) g (*P*=.03), respectively, whereas no significant reduction was observed in the fat (*P*=.12) or salt (*P*=.11) intake. The daily fiber intake significantly declined from 14.2 (IQR 12.6-16.7) to 12.1 (IQR 10.8-13.9) g (*P*=.001).

Changes in lifestyle based on the J-SDSCA are summarized in Table 4. The number of days the participants ate >300 g of vegetables significantly increased from 2.0 (IQR 1.0-3.0) to 4.0 (IQR 2.0-5.8) days per week (*P*<.001), whereas there was no change in the number of days per week the participants ate high-fat foods or the number of days per week their nutrient intake was distributed evenly throughout the day (eg, eating 3 well-balanced meals). The number of days they did at least 30 minutes of physical activity, including walking, increased significantly from 2.0 (IQR 1.0-3.0) to 3.0 (IQR 2.0-6.0) days per week (*P*<.001). In addition, the number of days per week they participated in a specific exercise session (such as swimming, walking, or biking) significantly increased (*P*=.02). As for the total scores, specific diet and exercise significantly improved from 7.0 (IQR 5.0-9.0) to 8.0 (IQR 6.0-10.8; *P*=.01) and from 2.5 (IQR 1.0-4.8) to 6.0 (IQR 3.0-7.0; *P*=.001), respectively.

The mean number of daily steps underwent no significant change between the first 7 days and last 7 days of using DialBeticsLite (8074, SD 3522 to 8700, SD 4240 steps; *P*=.32).

**Table 3:** Changes in the nutritional intake before and after the DialBeticsLite intervention.

	Baseline measurements (n=47), median (IQR)	Measurements after the intervention (n=47), median (IQR)	*P* values^a^
Energy (kcal/day)	1953 (1790-2395)	1877 (1690-2153)	.02
Protein (g/day)	71.4 (63.5-86.4)	66.4 (57.2-79.6)	.002
Lipid (g/day)	72.2 (58.6-91.8)	66.6 (54.7-80.7)	.12
Carbohydrate (g/day)	227.7 (198.4-277.6)	216.3 (181.6-250.5)	.03
Dietary fiber (g/day)	14.2 (12.6-16.7)	12.1 (10.8-13.9)	.001
Salt intake (g/day)	10.0 (8.4-11.5)	9.3 (8.5-10.4)	.11

^a^Wilcoxon signed-rank test for nonparametric variables.

**Table 4:** Changes in the J-SDSCA^a^ score before and after the DialBeticsLite intervention.

Questions			Baseline measurements (n=48), median (IQR)		Measurements after using DialBeticsLite (n=48), median (IQR)		*P* values^b^
**Specific diet**							
	On how many of the last 7 days did you eat approximately >300 g vegetables? (days per week)	2.0 (1.0-3.0)		4.0 (2.0-5.8)		<.001	
	On how many of the last 7 days did you eat high-fat foods such as red meat or full-fat dairy products? (days per week)	3.0 (2.0-4.0)		2.5 (2.0-4.0)		.22	
	On how many of the last 7 days did you distribute all nutrients evenly through the day? (days per week)	0.0 (0.0-2.75)		1.0 (0.0-3.0)		.23	
	Total score	7.0 (5.0-9.0)		8.0 (6.0-10.8)		.01	
**Exercise**							
	On how many of the last 7 days did you participate in at least 30 minutes of physical activity? (total minutes of continuous activity, including walking; days per week)	2.0 (1.0-3.0)		3.0 (2.0-6.0)		<.001	
	On how many of the last 7 days did you participate in a specific exercise session (such as swimming, walking, or biking) other than what you do around the house or as part of your work? (days per week)	1.0 (0.0-2.0)		1.5 (0.0-3.0)		.02	
	Total score	2.5 (1.0-4.8)		6.0 (3.0-7.0)		.001	

^a^J-SDSCA: Japanese-Translated Summary of Diabetes Self-Care Activities.

^b^Wilcoxon signed-rank test for nonparametric variables.

### Comparison of the Outcomes Between the 2 Companies

The use period of the system was different between the 2 companies; however, the reductions in BW (−3.9% for company X, 95% CI −7.7% to −1.5% vs −2.6% for company Y, 95% CI −7.3% to −0.9%); WC (−5.3% for company X, SD 4.6% vs −3.3% for company Y, SD 5.3%); and visceral fat area (−20.9% for company X, SD 14.2% vs −13.6% for company Y, SD 24%) at the end of the intervention were greater, although not significantly so, in company X (*P*=.46). The measurement rates of blood glucose before breakfast (88.1%, SD 25.8% for company X and 79.4%, SD 27.8% for company Y; *P*=.37); blood glucose at bedtime (81.6%, SD 24.3% for company X and 65.9%, SD 29.6% for company Y; *P*=.11); BW (97.2%, SD 4.9% for company X and 90.2%, SD 11% for company Y; *P*=.004); BP before breakfast (91.3%, SD 21.3% for company X and 82.9%, SD 17.8% for company Y; *P*=.15); BP at bedtime (80%, SD 24.1% for company X and 54.4%, SD 32% for company Y; *P*=.005); and daily steps (93.5%, SD 13% for company X and 91.3%, SD 17.9% for company Y; *P*=.66) were higher, especially for BW, among the participants from company X than among those from company Y in the first 2 months of the study.

### Usability of DialBeticsLite

The results of the usability survey are summarized in Table 5. The results showed that 85% (41/48) of the participants felt that using DialBeticsLite had improved their lifestyle and self-management skills. In total, 88% (42/48) of the participants responded that using the system and improving their lifestyle gave them a sense of security. On average, the participants spent 15.7 minutes per day using the system, and 88% (42/48) of them reported that the system was worth using for the amount of time they spent.

**Table 5:** Usability survey results.

Statement or question	Response	
	Yes, n (%)	Others, mean (SD)
1. I used the blood sugar monitor with no problem. (n=32)	24 (75)	N/A^a^
2. I used the sphygmomanometer with no problem. (n=48)	47 (98)	N/A
3. I used the pedometer with no problem. (n=48)	44 (92)	N/A
4. The interface of the system was easy to use. (n=47)	21 (45)	N/A
5. The instructions were easy to understand. (n=47)	45 (96)	N/A
6. The devices caused me physical discomfort. (n=48)	7 (15)	N/A
7. It was difficult to incorporate the system into daily practice. (n=48)	19 (40)	N/A
8. Any technical problems were resolved within 24 hours. (n=43)	22 (51)	N/A
9. Using the system and improving my lifestyle gave me a sense of security. (n=48)	42 (88)	N/A
10. I found the advice from the system useful. (n=46)	18 (39)	N/A
11. Participation in the study helped me to improve my lifestyle and self-management skills. (n=48)	41 (85)	N/A
12. Using the system took too much of my time. (n=48)	13 (27)	N/A
13. Using the system caused me some problems. (n=48)	13 (27)	N/A
14. How much time did you spend using the system? (minutes; n=48)	N/A	15.7 (8.0)
15. Is the system worth using for the time you spent? (n=48)	42 (88)	N/A

^a^N/A: not applicable.

## Discussion

### Principal Findings

The intervention using the ICT-based self-management system “DialBeticsLite” to support participants with AO yielded high continuous measurement rates, and 88% (42/48) of the participants reported that the system was worth using for the time they spent, demonstrating that DialBeticsLite is a feasible AO treatment solution (Table 5). After the intervention, which consisted of the initial 40-minute educational session and 2 to 3 months of use of DialBeticsLite, BW, BMI, WC, visceral fat area, systolic BP, and diastolic BP all improved (Table 2). The prevalence of AO reduced by 13% (6/42) in this sample (*P*=.03).

To the best of our knowledge, this is the first study to document the effects of a lifestyle intervention using an ICT-based self-management system on several parameters, including visceral fat area, in participants with AO. Previous studies have examined the effects of ICT-based systems in patients with obesity but focused on body mass without collecting data on body composition, such as visceral fat area [9,10]. Our results, which showed decreases in BW mediated by an ICT-based self-management system, are consistent with previous studies in patients with obesity, which showed a 1.9 to 4.8 kg decrease in BW or >5% reduction in the initial BW using other intervention methods for 3 months, such as internet-based programs [9,10]. In addition to decreases in BW, visceral fat area decreased significantly in this study, suggesting that a decrease in visceral fat area contributed to a decrease in BW. The daily energy intake also decreased, and the total score for diet as measured by the J-SDSCA increased significantly; it is presumed that the decrease in daily energy intake led to the loss of BW, BMI, WC, and visceral fat area. Greater reductions in BW, WC, and visceral fat area among participants from company X than in those from company Y may be attributed to the higher measurement rates in company X. Even though the HbA_1c_ levels of the participants were within the normal range at the study onset, HbA_1c_ was further reduced significantly after the intervention by 0.2%. This can be attributed to the overall effect of lifestyle changes and daily blood glucose monitoring supported by DialBeticsLite. DialBeticsLite incorporated feedback and monitoring as a behavior change technique, and a study had proven that automated personalized feedback through mHealth leads to lifestyle behavior change [22].

The measurement rates remained high over the study period (Table 1) compared with the previously reported studies in which ICT-based systems were introduced without any in-person interaction. Our previous study that was conducted entirely remotely using a self-management smartphone app for patients with type 2 diabetes and prediabetes showed a rapid decline in retention rates in the absence of intervention by medical professionals [23]. In this study, the in-person educational session on ICT system use by our research team for the study participants at baseline and reminders from our research team prompting participants to resume measurement when they stopped measurement for more than a week may have helped maintain better measurement and recording rates in this study. Relationships with health care providers have been shown to increase patients’ willingness to achieve goals [24], and our study showed that real-time feedback from the app coupled with targeted support from health care providers for patients who are prone to disengage from the use of mHealth is feasible, which leads to improved lifestyle habits and clinical outcomes. In addition, the recruitment of company employees may have created a sense of obligation among participants to complete the study, resulting in a better retention rate. Nevertheless, the short study duration in this study did not permit investigation of long-term retention and engagement with the intervention. To boost scalability to a wider network of users and reduce the burden on health care providers, unsupported mHealth may be necessary, which warrants the need to explore options of human-like features and experiences in the mHealth intervention, such as the use of chatbot and web-based coach [25].

The present findings regarding dietary intake suggest that more personalized feedback is necessary to improve the diet. The daily dietary fiber intake declined significantly (Table 3), presumably because of the decrease in the total dietary intake. Given that previous national health and nutrition examination surveys reported that the intake of dietary fiber declined along with decreased total energy intake [26], it is difficult to increase the intake of fiber under restricted total energy intake. In addition, the daily salt intake of the study participants was 9.3 g per day after the intervention (Table 3), which is still very high compared with the recommendation of a maximum intake of 6 g per day for adults by the Japanese Society of Hypertension [27]. Providing meaningful information to the users through the intervention about salt or dietary fiber intake is expected to increase their awareness of these detrimental behaviors. The current intervention provides the same automated feedback to the users regardless of their understanding of the intake levels and dietary sources of each nutrient. Therefore, the improvement of automatic feedback such that it is tailored to match the levels of each user’s knowledge and interest in diet and nutrition should be considered in the future.

### Limitations

Several limitations to the present findings have to be considered. First, this was a small pilot study with 48 participants with the mean age of 46.8 (SD 6.8; range 32-62) years, and most participants were male (n=44, 92%). The gender distribution corresponded to the prevalence of metabolic syndrome according to the criteria set by the Japanese Committee of the Criteria for Metabolic Syndrome, which is significantly higher in men than in women (12.1% vs 1.7%) [28]. Moreover, the higher affinity for ICT use among men may have partly contributed to the gender disparity in our study population [29].

Second, the duration of intervention by the system was short (61 and 93 days); therefore, we were unable to assess the long-term effects and use patterns of the system. Third, in this study, it was not possible to determine whether the improvements in physical parameters such as BW, BMI, WC, visceral fat area, and systolic and diastolic BP were the effects of “DialBeticsLite” itself; the educational session at baseline might have affected the subjects’ behavior. Nevertheless, given the fact that all participants reported being motivated by the sense of security using the system imparted, there is a strong possibility that using the system raised their awareness of newer methods of self-management based on ICT.

As data from 2 groups (participants from company X and those from company Y) were collected from different periods and seasons, there is a possibility of bias, as the frequency of walking activities varied according to the outdoor temperature (step counts) and seasonal variation may influence BP and dietary intake. This was unavoidable, as both companies had different time availabilities to participate in the study owing to different job natures.

We also could not avoid potential recall bias and input of socially acceptable information when participants self-reported their dietary record; nevertheless, we tried to reduce the bias by instructing patients to report their diet immediately after each meal. At the time of the study, no validated questionnaire was available in the literature to test the usability of this mHealth app, and we sought to reduce the bias by establishing the face validity of the questionnaire.

Finally, this study adopted a pre-post study design, which may be subject to other confounders; thus, the results should be interpreted with caution. The participants were those who were willing to participate in the study and were not randomly chosen. Considering these limitations, the study findings must be validated in a larger randomized controlled trial with a longer duration, and we are currently conducting a randomized controlled trial to examine whether DialBeticsLite is effective in improving parameters related to metabolic syndromes.

### Conclusions

In conclusion, DialBeticsLite was shown to be a feasible and potentially effective tool for improving AO by providing patients with real-time support based on their measurements and inputs of BW, BP, pedometer count, blood glucose, exercise, and dietary intake. Preliminary findings showed that DialBeticsLite significantly reduced the BW, BMI, WC, and visceral fat area of patients with AO. A larger randomized controlled trial with a longer duration to further validate these findings is in progress.

## Appendix

Multimedia Appendix 1 An overview of a feedback report from health care providers to the users of DialBeticsLite. Under “measurement data,” the percentages shown represent the average measurement rate over the intervention period. Under “achievement status of treatment goal,” the percentages shown represent the achievement rate of target blood glucose, blood pressure, and pedometer count.” Data are presented as mean (SD) or median (IQR).

## Abbreviations

AO: abdominal obesity
BP: blood pressure
BW: body weight
HbA_1c_: hemoglobin A_1c_
ICT: information and communication technology
J-SDSCA: Japanese-Translated Summary of Diabetes Self-Care Activities
mHealth: mobile health
WC: waist circumference

## References

[1] Afshin A, Forouzanfar MH, Reitsma MB, Sur P, Estep K, Lee A, Marczak L, Mokdad AH, Moradi-Lakeh M, Naghavi M, Salama JS, Vos T, Abate KH, Abbafati C, Ahmed MB, Al-Aly Z, Alkerwi A, Al-Raddadi R, Amare AT, Amberbir A, Amegah AK, Amini E, Amrock SM, Anjana RM, Ärnlöv J, Asayesh H, Banerjee A, Barac A, Baye E, Bennett DA, Beyene AS, Biadgilign S, Biryukov S, Bjertness E, Boneya DJ, Campos-Nonato I, Carrero JJ, Cecilio P, Cercy K, Ciobanu LG, Cornaby L, Damtew SA, Dandona L, Dandona R, Dharmaratne SD, Duncan BB, Eshrati B, Esteghamati A, Feigin VL, Fernandes JC, Fürst T, Gebrehiwot TT, Gold A, Gona PN, Goto A, Habtewold TD, Hadush KT, Hafezi-Nejad N, Hay SI, Horino M, Islami F, Kamal R, Kasaeian A, Katikireddi SV, Kengne AP, Kesavachandran CN, Khader YS, Khang Y, Khubchandani J, Kim D, Kim YJ, Kinfu Y, Kosen S, Ku T, Defo BK, Kumar GA, Larson HJ, Leinsalu M, Liang X, Lim SS, Liu P, Lopez AD, Lozano R, Majeed A, Malekzadeh R, Malta DC, Mazidi M, McAlinden C, McGarvey ST, Mengistu DT, Mensah GA, Mensink GB, Mezgebe HB, Mirrakhimov EM, Mueller UO, Noubiap JJ, Obermeyer CM, Ogbo FA, Owolabi MO, Patton GC, Pourmalek F, Qorbani M, Rafay A, Rai RK, Ranabhat CL, Reinig N, Safiri S, Salomon JA, Sanabria JR, Santos IS, Sartorius B, Sawhney M, Schmidhuber J, Schutte AE, Schmidt MI, Sepanlou SG, Shamsizadeh M, Sheikhbahaei S, Shin M, Shiri R, Shiue I, Roba HS, Silva DA, Silverberg JI, Singh JA, Stranges S, Swaminathan S, Tabarés-Seisdedos R, Tadese F, Tedla BA, Tegegne BS, Terkawi AS, Thakur JS, Tonelli M, Topor-Madry R, Tyrovolas S, Ukwaja KN, Uthman OA, Vaezghasemi M, Vasankari T, Vlassov VV, Vollset SE, Weiderpass E, Werdecker A, Wesana J, Westerman R, Yano Y, Yonemoto N, Yonga G, Zaidi Z, Zenebe ZM, Zipkin B, Murray CJ, GBD 2015 Obesity Collaborators (2017). Health effects of overweight and obesity in 195 countries over 25 years. N Engl J Med.

[2] Gremeaux V, Drigny J, Nigam A, Juneau M, Guilbeault V, Latour E, Gayda M (2012). Long-term lifestyle intervention with optimized high-intensity interval training improves body composition, cardiometabolic risk, and exercise parameters in patients with abdominal obesity. Am J Phys Med Rehabil.

[3] Ilanne-Parikka P, Eriksson JG, Lindström J, Peltonen M, Aunola S, Hämäläinen H, Keinänen-Kiukaanniemi S, Laakso M, Valle TT, Lahtela J, Uusitupa M, Tuomilehto J, Finnish Diabetes Prevention Study Group (2008). Effect of lifestyle intervention on the occurrence of metabolic syndrome and its components in the Finnish Diabetes Prevention Study. Diabetes Care.

[4] Nanri A, Tomita K, Matsushita Y, Ichikawa F, Yamamoto M, Nagafuchi Y, Kakumoto Y, Mizoue T (2012). Effect of six months lifestyle intervention in Japanese men with metabolic syndrome: randomized controlled trial. J Occup Health.

[5] Gold BC, Burke S, Pintauro S, Buzzell P, Harvey-Berino J (2007). Weight loss on the Web: a pilot study comparing a structured behavioral intervention to a commercial program. Obesity (Silver Spring).

[6] Arem H, Irwin M (2011). A review of Web-based weight loss interventions in adults. Obes Rev.

[7] Dalle Grave R, Calugi S, El Ghoch M (2013). Lifestyle modification in the management of obesity: achievements and challenges. Eat Weight Disord.

[8] Dalle Grave R, Centis E, Marzocchi R, El Ghoch M, Marchesini G (2013). Major factors for facilitating change in behavioral strategies to reduce obesity. Psychol Res Behav Manag.

[9] Coons MJ, Demott A, Buscemi J, Duncan JM, Pellegrini CA, Steglitz J, Pictor A, Spring B (2012). Technology interventions to curb obesity: a systematic review of the current literature. Curr Cardiovasc Risk Rep.

[10] Saperstein SL, Atkinson NL, Gold RS (2007). The impact of Internet use for weight loss. Obes Rev.

[11] Waki K, Fujita H, Uchimura Y, Omae K, Aramaki E, Kato S, Lee H, Kobayashi H, Kadowaki T, Ohe K (2014). DialBetics: a novel smartphone-based self-management support system for type 2 diabetes patients. J Diabetes Sci Technol.

[12] Park SH, Hwang J, Choi YK (2019). Effect of mobile health on obese adults: a systematic review and meta-analysis. Healthc Inform Res.

[13] Seo DC, Niu J (2015). Evaluation of Internet-based interventions on waist circumference reduction: a meta-analysis. J Med Internet Res.

[14] Després JP, Lemieux I (2006). Abdominal obesity and metabolic syndrome. Nature.

[15] Sim J, Lewis M (2012). The size of a pilot study for a clinical trial should be calculated in relation to considerations of precision and efficiency. J Clin Epidemiol.

[16] Committee to Evaluate Diagnostic Standards for Metabolic Syndrome (2005). [Definition and the diagnostic standard for metabolic syndrome--Committee to Evaluate Diagnostic Standards for Metabolic Syndrome]. Nihon Naika Gakkai Zasshi.

[17] (2020). Japanese Clinical Practice Guideline for Diabetes 2019. The Japan Diabetes Society.

[18] Japanese official physical guidelines for health promotion. Ministry of Health, Labor and Welfare.

[19] Shiga T, Hamaguchi Y, Oshima H, Kanai M, Hirata K, Hosoda K, Nakao K (2009). A new simple measurement system of visceral fat accumulation by bioelectrical impedance analysis. Proceedings of World Congress on Medical Physics and Biomedical Engineering.

[20] Omura-Ohata Y, Son C, Makino H, Koezuka R, Tochiya M, Tamanaha T, Kishimoto I, Hosoda K (2019). Efficacy of visceral fat estimation by dual bioelectrical impedance analysis in detecting cardiovascular risk factors in patients with type 2 diabetes. Cardiovasc Diabetol.

[21] Toobert DJ, Hampson SE, Glasgow RE (2000). The summary of diabetes self-care activities measure: results from 7 studies and a revised scale. Diabetes Care.

[22] Rabbi M, Pfammatter A, Zhang M, Spring B, Choudhury T (2015). Automated personalized feedback for physical activity and dietary behavior change with mobile phones: a randomized controlled trial on adults. JMIR Mhealth Uhealth.

[23] Yamaguchi S, Waki K, Nannya Y, Nangaku M, Kadowaki T, Ohe K (2019). Usage patterns of GlucoNote, a self-management smartphone app, based on ResearchKit for patients with type 2 diabetes and prediabetes. JMIR Mhealth Uhealth.

[24] Selter A, Tsangouri C, Ali SB, Freed D, Vatchinsky A, Kizer J, Sahuguet A, Vojta D, Vad V, Pollak J, Estrin D (2018). An mHealth app for self-management of chronic lower back pain (Limbr): pilot study. JMIR Mhealth Uhealth.

[25] Müssener U (2021). Digital encounters: human interactions in mHealth behavior change interventions. Digit Health.

[26] Japanese National Health and Nutrition Survey in 2017. Ministry of Health, Labor and Welfare.

[27] Recommendations from Salt Reduction Committee, Japanese Society of Hypertension. The Japanese Society of Hypertension.

[28] Arai H, Yamamoto A, Matsuzawa Y, Saito Y, Yamada N, Oikawa S, Mabuchi H, Teramoto T, Sasaki J, Nakaya N, Itakura H, Ishikawa Y, Ouchi Y, Horibe H, Shirahashi N, Kita T (2006). Prevalence of metabolic syndrome in the general Japanese population in 2000. J Atheroscler Thromb.

[29] Nasah A, DaCosta B, Kinsell C, Seok S (2010). The digital literacy debate: an investigation of digital propensity and information and communication technology. Educ Tech Res Dev.

