# Supplementary figures and images for: The Use of Information and Communication Technology–Based Self-management System DialBeticsLite in Treating Abdominal Obesity in Japanese Office Workers: Prospective Single-Arm Pilot Intervention Study
Source: JMIR Diabetes. 2022 Nov 28;7(4):e40366. doi: 10.2196/40366 (PMC9745649; doi:10.2196/40366)

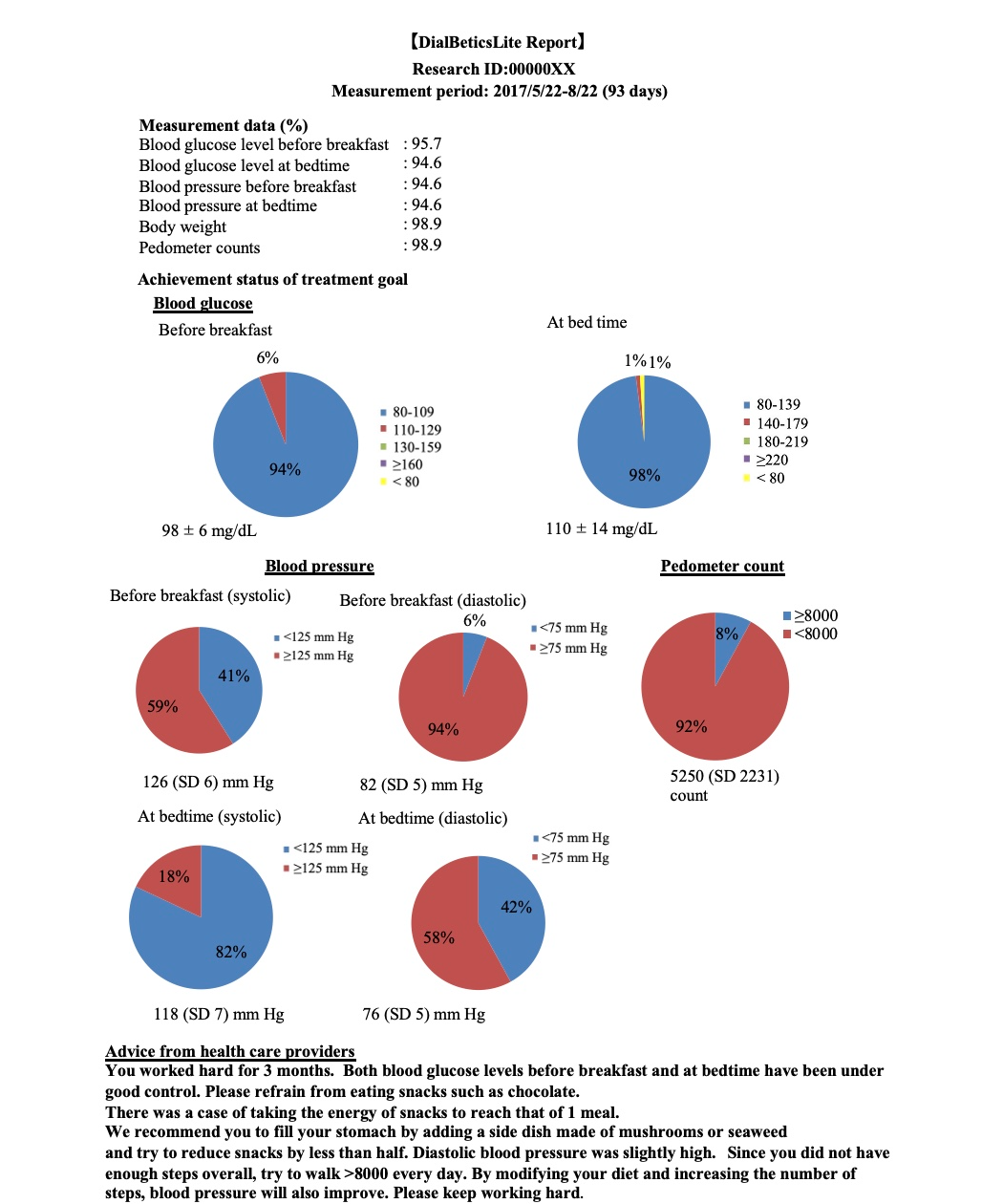

Supplement: Multimedia Appendix 1 [file diabetes_v7i4e40366_app1.png]
